# Effect of a novel telehealth device for dietary cognitive behavioral intervention in overweight or obesity care

**DOI:** 10.1038/s41598-023-33238-4

**Published:** 2023-04-20

**Authors:** Yi-Ya Fang, Jia-In Lee, Nai-Yuan Wu, Chiao-I Chang, Meng-Chuan Huang, Chun-Ying Lee, Jui-Yen Huang, Gwo Giun Chris Lee, Cheng-Sheng Chen

**Affiliations:** 1 Graduate Institute of Medicine, College of Medicine, Kaohsiung Medical University, Kaohsiung, Taiwan; 2 Department of Psychiatry, Kaohsiung Medical University Hospital, Kaohsiung Medical University, Kaohsiung, Taiwan; 3 Institute of Biomedical Informatics, National Yang Ming Chiao Tung University, Hsinchu, Taiwan; 4 Department of Public Health and Environmental Medicine, College of Medicine, Kaohsiung Medical University, Kaohsiung, Taiwan; 5 Division of Nutrition and Dietetics, Kaohsiung Medical University Hospital, Kaohsiung Medical University, Kaohsiung, Taiwan; 6 Division of Family Medicine, Kaohsiung Medical University Hospital, Kaohsiung Medical University, Kaohsiung, Taiwan; 7 Department of Pediatrics, Kaohsiung Medical University Hospital, Kaohsiung Medical University, Kaohsiung, Taiwan; 8 Department of Electrical Engineering, National Cheng Kung University, Tainan, Taiwan

**Keywords:** Biotechnology, Health care

## Abstract

Obesity has become a major public health issue which relate to numerous physical problems and highly comorbid with depression and anxiety. Recently, some studies of technology-based interventions for weight reduction emerged to overcome the barriers from time, cost and distance. Mood component and eating behavior related to obesity are less discussed so far with technology-based intervention though. This pilot study was aimed to investigate the effect of telehealth assisted intervention on weight reduction, mood status, and eating behavior change under a smartphone application (app) with novel 3D food picture recognition and incorporated with cognitive behavioral training programs. Adult aged 30–60 years old with overweight were recruited and randomly assigned to control-first group and intervention-first group. In period 1, control-first group had regular life and intervention-first group underwent app intervention; in period 2, two groups went crossover. Body composition and psychological/behavioral questionnaires were collected at baseline, end of period 1, and end of period 2. Nonparametric statistics was performed for data analyzing. A total of 20 participants were enrolled. In control-first group, there were statistically significant reduction in body weight (− 0.55 kg, *p* = 0.02) and change of body weight percentage (− 0.6%, *p* = 0.02) after App use. In intervention-first group, the fat percentage decreased by 0.4% after App use in period 1, and increased by 0.05% in period 2. The integrated crossover data revealed that subjects of App group had significant improvements in mindful eating behavior. This pilot study showed the effectiveness in using CogniNU app for weight control and eating behavior. The difference of short-term and long-term effectiveness of technology-based weight control intervention deserves more investigation in the future.

*Clinical Trial Registration*: ISRCTN16082909.

## Introduction

Over these decades, significant rising of overweight and obesity prevalence draws major attention to the public health issues. Obesity rate has tripled since 1975 according to the World Health Organization (WHO) report. WHO also revealed that around two fifths of the population who aged over 18 years old was overweight in 2016. Obesity is confirmed to have strong relationship to numerous physical problems including hypertension, cardiovascular diseases, diabetes, musculoskeletal disorders and some cancers, contributing to decreased quality of life, disability and even premature death^1^.

It is also known that obesity is highly comorbid with depression and anxiety^2^. The relationship of obesity and mood disorder seems to be bi-directional^3^. Emotional eating means that someone manages negative or positive emotion by food intakes and often leads to overeating and obesity^4^. Psychological and behavioral interventions play crucial parts in weight reduction which might lead to better nutrition intake, medical adherence and physical activities^5^. Psychological and behavioral interventions are well-structured, organized and consist of multi-components such as cognitive behavioral therapy, acceptance therapy, and motivational interviewing. A previous review suggests that these interventions are usually provided by experienced professionals once a week in person with ranges of 4–6 months initially, and then followed by bi-weekly sessions^6^. However, this kind of high-intensity setting might hinder some people with obesity from receiving assistance.

Recently, some studies of technology-based interventions for weight control programs emerged to overcome the barriers from time, cost and distance. Past researches revealed promising effects for remarkable weight loss in patients with obesity owing to better adherence and easier self-monitoring^7^. The majority of these interventions were conducted via smartphone, for examples, mobile app, telephone calls and/or SMS, to either monitor weight change, daily intake, and/or physical activity. Some of these also provided feedback to participants via text messages or SMS, emails, or phone calls^8–10^. However, mood component and eating behavior related to obesity are less discussed so far with technology-based intervention though. A novel technology intervention called CogniNU App (previously called CogniAI) was developed to provide a more comprehensive program for individual with overweight or obesity (©2021 CogniNU Lab, all rights reserved.). This online artificial intelligence (AI) mindful eating program combined three disciplines: artificial intelligence (AI), psychiatry, and nutritional science. The CogniNU app utilized AI technology to analyze food images, to accurately identify food ingredients and nutrient contents. The app also provided digital program that contains concept of cognitive behavioral therapy (CBT) to help users identify unhealthy eating habits and behaviors and build knowledge about healthy eating, several sets of healthy low-fat or low-carbohydrate recipes for weight loss were also provided.

Therefore, this pilot study aimed to present the design of a smartphone application (app) CogniNU app and evaluate the effect of CogniNU on weight reduction, eating behavior, and mood.

## Materials and methods

### Participants and study design

This single-center prospective, randomized, crossover pilot study (ISRCTN16082909, registration date: 11/11/2022) was ethically approved by the Institutional Review Board at Kaohsiung Medical University Hospital (KMUHIRB-E[I]-20210067). Recruitment advertisements were released through the hospital intranet system to all employees. All enrolled participants provided written inform consents before participation. Included participants were adults aged between 30 and 60 years with a body mass index (BMI) ≥ 25 kg/m^2^. Exclusion criteria were major neuropsychiatric comorbidities, for examples, schizophrenia, major depressive disorders, stroke, epilepsy, and Parkinson’s disease.

**Figure 1:**
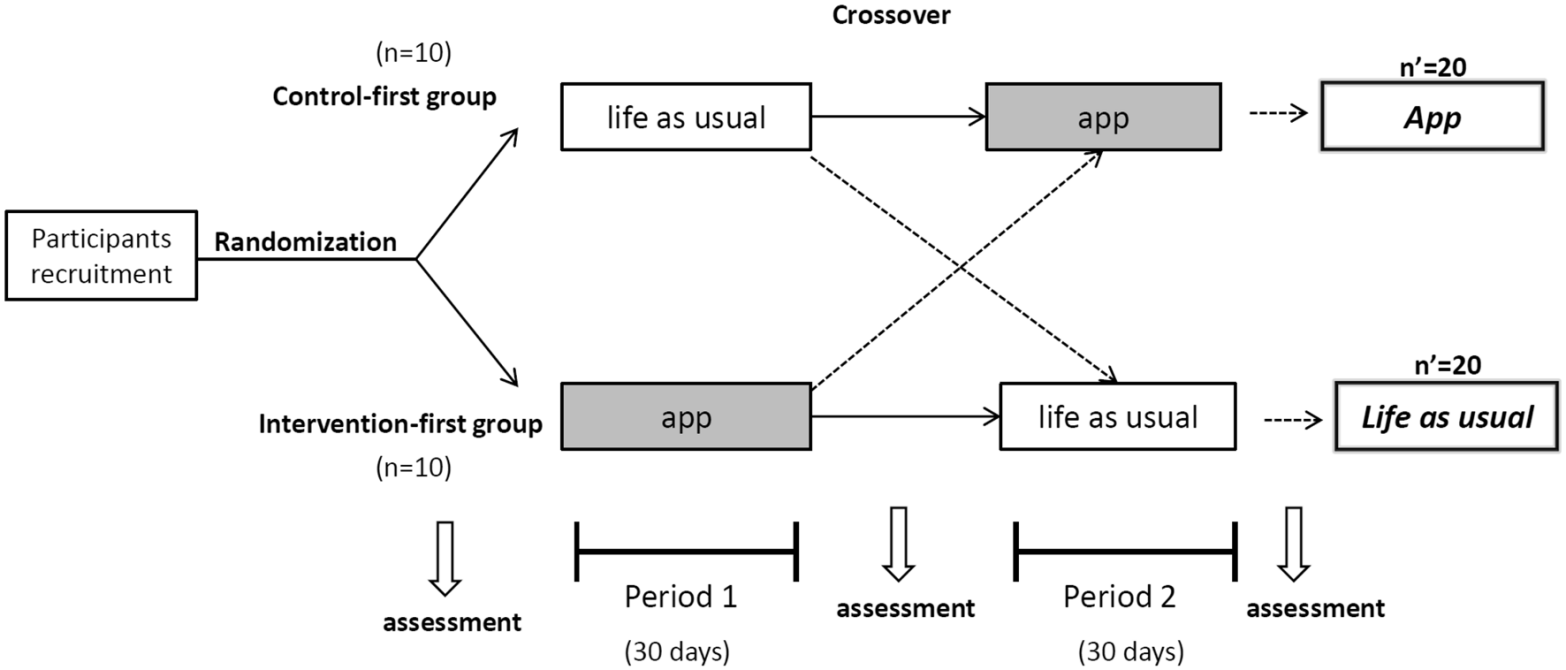
Randomization and flow of participants from baseline through day 60.

As illustrated in Fig. 1, all eligible participants were randomly assigned (1:1) to the control-first group or intervention-first group accordingly to their order of entry into the study. The study investigator, research coordinator, and staff assessing outcomes were blinded to the intervention allocation. The participants in intervention-first group were instructed to join an online AI mindful eating program and those in control-first group remained their usual lifestyle (period 1). After 30 days of intervention, all participants were switched to the opposite group in a crossover manner for the following 30-day intervention (period 2). All participants were evaluated at baseline, before crossover (day 30, the end of period 1), and after all interventions (day 60, the end of period 2). Body composition was measured at Kaohsiung Medical University Hospital and other assessments were collected via online survey.

### Application design

#### The integration of three disciplines

Supplementary Fig. 1 depicted the integration of three disciplines in the CogniNU app. The digital program based on the concept of CBT, modified from a 42-day weight loss program^11^, was linked with the nutrient database and output data from the AI analysis. The lower path in Supplementary Fig. 1illustrated the pipeline of AI technology which was designed to analyze food images for identifying ingredients and nutrients. The elements of AI technology included algorithm, big data, and computing.

#### Algorithm

Food images taken from smartphone were initially processed by the ‘AI food image recognition’, which was lightweight deep learning algorithmic software based on proprietary machine learning techniques^12–16^. Then the ‘ingredient categorization’, a deep analytics algorithm, decomposed the images, identified the food contents, and categorized food contents by ingredients. The following ‘nutrition guestimation’ analyzed the nutrients of ingredients per serving (100 g) according to the Food composition database in Taiwan^17^.

#### Big data

##### Construction of the intelligent food image database

The Intelligent Food Image Database (IFID) was built up by collecting food images from nine open source datasets (Supplementary Table 1). The IFID comprised more than 370,000 food images of Asian and Western meals. These images were pre-processed with techniques of auto-white balancing and contrast limited adaptive histogram equalization to enhance image color, contrast, and intensity^18^. Pre-processing was performed to ensure better recognition accuracy in different situations, such as images obtained in dim light or under less than ideal conditions.

##### Labeling food images

Food images were labeled according to their ingredient categories (Supplementary Table 2). Contextual data and dish names of food images which were extracted by machine learning based methods were incorporated into the IFID during image labeling. Based on the concept of contextual information, relevant data were stored closely together, which would allow high-speed data access and further improve recognition accuracy^19,20^. Distribution of food images in the IFID by ingredient category was displayed in Supplementary Fig. 2.

#### Computing

A lightweight and low-complexity algorithm for smartphone was developed with the employment of the proprietary analytics architecture technologies which are based on algorithm-architecture co-design. This algorithm provided high-speed deep learning training and near real-time testing^21,22^. These features facilitated the future development of a complete health and wellness ecosystem to fostering healthcare-on-demand.

### Outcome measurement

At baseline, the end of period 1 (day 30), and the end of period 2 (day 60), all participants received body composition measurement and were requested to complete self-assessment questionnaires for eating behavior, mood assessment, quality of life, physical activity, and sleep quality.

#### Body composition

Measures of body composition included body weight, BMI, muscle mass, fat mass, body fat percentage, and visceral fat area were obtained using the InBody 770 (InBodyUSA, Cerritos, CA). The device utilizes hand-to-foot bioelectrical impedance analysis to send alternating currents of different frequencies through the body and combines the impedance values and inputs of age, sex, and body height to predict body compositions.

#### Behavioral and psychosocial outcomes

##### Eating behavior

The Dietary Behavior Questionnaire (DBQ) and Mindful Eating Behavior Scale (MEBS) were used to evaluate eating behaviors.

The DBQ contains 12 questions and each question is scored on a 4-point scale (0 = rarely, 1 = occasionally, 2 = often, 3 = always). Question 5 to 12 is reversely scored. The total score ranges from 0 to 36 and a higher total score indicates better eating behavior^23^.

The MEBS contains 17 questions and four domains (focused eating, hunger and satiety cues, eating with awareness, and eating without distraction). Each question is scored on a 5-point Likert scale (1 = never, 2 = rarely, 3 = occasionally, 4 = often, 5 = always), and question 11 to 17 were reversely scored. A higher score indicates more mindful eating^24^.

##### Mood assessment

The five-item Brief Symptom Rating Scale (BSRS-5) and Chinese version of Beck Depression Inventory II (BDI-II) were used for mood assessment.

The BSRS-5 consists of five questions and one additional question (suicidal thoughts) to identify common psychopathology, including insomnia, anxiety, anger, depression, and low self-esteem. It is a self-report scale that can be used as a tool for screening psychiatric morbidity^25,26^. Each item is scored on a severity scale from 0 to 4 for the severity of subjectively perceived distress (0 = not at all, 1 = a little bit, 2 = moderately, 3 = quite a bit, 4 = extremely). A total score ≥ 15 or a score ≥ 2 on the additional question for suicidal thoughts may indicate severe mood disorder^25,26^.

The BDI-II, developed by Beck et al. in 1961 and revised in 1996 as current version, contains 21 questions to assess depressive symptoms and their severity^27,28^. Each question is scored on a severity scale from 0 to 3. The normal range for the Chinese version of BDI-II is 0–16, 17–22 for mild depression, 23–30 for moderate depression, and 31–63 for severe depression^27,29^.

##### Others

Quality of Life was measured via WHO Quality-of-Life scale (WHOQOL-BREF)- Taiwan version. Divided into four aspects, (1) Physiological aspect (including physiology and degree of independence); (2) Psychological aspect; (3) Social relationship aspect; (4) Environmental scope. Each question in the questionnaire is scored using the 5-point Likert Scale, with higher scores indicating better quality of life^30^. The Taiwan version of International Physical Activity Questionnaire Short Form (IPAQ-SF) was used to assess metabolic equivalent (MET) in a week. The IPAQ-SF includes seven questions to record the frequency and duration of four different levels of physical activities in the past one week, including vigorous activity, moderate activity, walking, and sitting^31^. The Pittsburgh Sleep Quality Index (PSQI), developed by Buysee et al. in 1989, consists of ten self-assessed sleep questions. The total PSQI score ranges from o to 21. A total PSQI score ≥ 5 indicates poor sleep, with the higher score associated with poorer sleep quality^32^.

### Sample size and Statistical analysis

This was a pilot study that we tried to recruit at least 20 participants to perform regression analysis. Descriptive analysis was used for demographic data and basic distribution of each indicator. For comparisons of continuous variables, Wilcoxon rank sum test was used for between-group comparisons and Wilcoxon matched-paired sign-rank test for within-group comparisons. Fisher’s exact test was performed for comparisons of dichotomous variables. All analyses were performed using STATA® 16.1 (StataCorp LLC, Texas, USA). A two-tailed *p* value < 0.05 indicates statistical significance.

### Ethics statement

This prospective clinical pilot study protocol was approved by the ethic committee of the Institutional Review Board at Kaohsiung Medical University Hospital (KMUHIRB-E[I]-20210067). All methods were performed in accordance with relevant guidelines and regulations and informed consent was obtained from each subject.

## Results

This study enrolled 20 participants with a median age of 42.0 years (IQR = 36–47.5) and 75% of them were female. Median BMI was 27.9 kg/m^2^ (IQR = 25.9–33.3). As presented in Table 1, there was no significant difference in demographics and baseline characteristics between the control-first and intervention-first group (Table 1).

**Table 1:** Demographics and baseline characteristics of participants.

	Control-first group (n = 10)	Intervention-first group(n = 10)	Total (n = 20)	Between group comparison
				*P* value
Age	38.5 (35–46)	44 (37–49)	42 (36–47.5)	0.38
Sex (female)*	7 (70)	8 (80)	15 (75)	1.00
Body composition				
Body weight	82.2 (63.2–101.4)	75.4 (70.5 88.9)	76.7 (67.7–89)	0.76
BMI	28.1 (25.8–34.7)	27.5 (26.1–29.3)	27.9 (25.9–33.3)	0.42
Muscle weight (kg)	27.2 (22–30.3)	25.7 (23.7–27.7)	26.2 (23.1–30)	0.79
Fat mass (kg)	29.1 (24.1–37.6)	26.7 (25.4–28.5)	27.8 (25.4–36.6)	0.78
Fat percentage (%)	36.9 (35.5–42.7)	37.6 (33.7–41.2)	37.6 (35.5–42)	0.52
Visceral fat (cm^2^)	133.7 (122.1–178.8)	130 (113.5–138)	131.4 (117.3–172.5)	0.50
DBQ	20.5 (18–24)	21 (20–24)	21 (18.5–24)	0.64
MEBS				
Total scores	56 (53–60)	54 (48–56)	55.5 (51–57)	0.19
Focusing eating	20 (19–21)	18.5 (16–20)	19.5 (18–20.5)	0.14
Eating with awareness	6 (5–8)	7 (6–8)	6.5 (5.5–8)	0.49
Eating in response to hunger and satiety cues	17.5 (14–19)	16 (14–17)	16 (14–18.5)	0.42
Eating without distraction	12.5 (11–15)	12.5 (11–15)	12.5 (11–15)	0.85
Mood				
BSRS-5	10 (8–12)	9.5 (7–10)	10 (7.5–10.5)	0.20
BDI-II	5.5 (3–11)	10 (3–14)	8 (3–13.5)	0.73
PSQI	6 (5–9)	6 (5–8)	6 (5–8)	0.82
IPAQ-SF (MET/week)	348.5 (66–594)	1116.5 (259–1988)	495.5 (66–1427)	0.09
WHOQOL				
Total scores	14.3 (12.6–15.6)	14.6 (13.6–16.7)	14.6 (12.9–15.9)	0.68
Physiological aspect	15.7 (13–17)	15.7 (14.3–17.7)	15.7 (14–17.4)	0.97
Psychological aspect	15 (12.7–16.7)	16 (14.7–17.3)	15.7 (14–17)	0.70
Social relationship	14.5 (12–16)	15 (14–17)	15 (12.5–16)	0.54
Environment scope	16 (14–17.8)	16 (14.7–19.1)	16 (14.2–17.8)	0.59

Data were presented as median (interquartile range) or count (percentage)*.

*DBQ* Dietary Behavior Questionnaire; *MEBS* Mindful Eating Behavior Scale; BSRS-5, five-item Brief Symptoms Rating Scale; *BDI-II* Beck Depression Inventory II; *PSQI* Pittsburgh Sleep Quality Index; *IPAQ-SF* International Physical Activity Questionnaire Short Form; *MET* metabolic equivalent; *WHOQOL* WHO quality of life.

Subjects of intervention-first group had decrease in body fat percentage by 0.4% in the intervention period and increase by 0.05% in the control period, achieving significant difference in changes in body fat percentage between the two periods (*p* = 0.01). As for the control-first group, the changes in body weight (kg) in the two periods were also significantly different (− 0.6 kg in the intervention period vs. + 0.8 kg in the control period, *p* = 0.02); the same results were also observed on body weight change percentage (%) (− 0.6% in the intervention period vs. + 0.7% in the control period, *p* = 0.02) (Table 2).

**Table 2:** Within-group and between group comparison of outcomes in different intervention period.

	Control-first group (n = 10)			Intervention-first group (n = 10)			Between group comparison	Between group comparison
	Period 1 (Control)	Period 2 (Intervention)		Period 1 (Intervention)	Period 2 (Control)		Period 1	Period 2
	Δ_1_	Δ_2_	*p* Value	Δ_1_	Δ_2_	*p* Value	*p* Value	*p* Value
Body weight (kg)	0.8 (−0.2, 1.1)	−0.6 (−0.9, 0.7)	*0.02	−0.1 (−0.5, 0.3)	−0.15 (−0.9, 0)	0.76	0.11	0.79
BMI	0.25 (−0.01, 0.4)	−0.25 (−0.3, 0.3)	0.15	−0.05 (−0.1, 0.2)	−0.05 (−0.3, 0)	0.61	0.615	0.76
Muscle weight (kg)	0.2 (−0.5, 0.9)	−0.2 (−0.5, -0.1)	0.17	−0.05 (−0.3, 0.6)	0 (−0.4, 0.1)	0.24	0.76	0.38
Fat mass (kg)	0.05 (−0.8, 1.4)	0.05 (−0.6, 1)	0.80	−0.4 (−1.2, 0.1)	0 (−0.5, 0.7)	0.11	0.34	0.91
Fat percentage (%)	0.1 (−1.2, 1.1)	0.25 (−0.7, 0.8)	0.61	−0.4 (−1.1, 0.1)	0.05 (−0.4, 0.8)	*0.01	0.62	0.69
Visceral fat (cm^2^)	−0.65 (−4.3, 8.3)	1.85 (−2.1, 4.4)	0.88	−0.8 (−4.8, 1.9)	0.75 (−1.8, 4.1)	0.65	0.70	1.00
Body weight change (%)	0.7 (−0.23, 1.3)	−0.6 (−1.1, 0.7)	*0.02	−0.13 (−0.6, 0.4)	−0.2 (−1, 0)	0.72	0.10	0.88
DBQ	−0.5 (−2, 2)	−0.5 (−2, 1)	0.76	1.5 (−1.5, 3)	1.5 (−2, 3.5)	0.94	0.50	0.59
MEBS								
Total scores	−2.5 (−4, 1)	2.5 (−1, 5)	0.17	0.5 (−2.5, 3.5)	−0.5 (−4, 1)	0.53	0.35	0.13
Focusing eating	−0.5 (−1, 1)	−0.5 (−4, 0)	0.57	0.5 (−1.5, 1.5)	0 (−1, 1)	0.72	0.72	0.26
Eating with awareness	0 (−1, 1)	0 (0, 1)	0.25	0 (−0.5, 1.5)	−0.5 (−1.5, 0.5)	0.35	0.65	0.11
Eating in response to hunger and satiety cues	0 (−1, 0)	0.5 (0, 2)	0.06	0 (0, 1)	−0.5 (−2, 1)	0.23	0.21	0.12
Eating without distraction	−1 (−3, 0)	0.5 (0, 1)	0.26	−1 (−2, –0.5)	− 1 (−2, 1)	0.32	0.65	0.30
BSRS-5	0 (−3, 0)	1 (0, 1)	0.23	0.5 (−0.5, 1)	0.5 (−1, 2)	0.57	0.32	0.85
BDI-II	−0.5 (− 3, 5)	0 (−1, 2)	0.68	−4.5 (−6.5, 0.5)	1 (−1.5, 3.5)	0.53	0.13	0.65
PSQI	0 (−1, 1)	0 (−1, 1)	0.72	0 (−2, 0)	2 (0, 2)	0.08	0.33	0.09
IPAQ-SF (MET/week)	−33 (−405, 80)	53 (−40, 252)	0.28	−906 (−198.4, 83.5)	−100 (−182.5, 56.5)	0.16	0.23	0.27
WHOQOL								
Total scores	−0.14(−0.57, 1)	0.35 (0, 1.1)	0.44	−0.35 (−0.9, 0.35)	0.3 (− 0.07, 0.9)	0.33	0.45	0.72
Physiological aspect	0 (−0.6, 1.1)	0 (−1.1, 0.6)	0.92	0.6 (−0.6, 1.4)	0.3 (0, 0.6)	0.73	0.69	0.62
Psychological aspect	−0.67 (−0.67, 0.67)	0.67 (0, 2)	0.22	−0.33 (−1.3, 1.3)	0.33 (−1.6, 1.3)	0.94	0.69	0.33
Social relationship	−0.5 (−1, 1)	0 (−1, 2)	0.57	−1 (−2, 0.5)	0.5 (−0.5, 2)	0.23	0.36	0.79
Environment scope	0.2 (−0.4, 0.8)	0.4 (0, 1.3)	0.51	−0.2 (−0.7, 0.4)	0 (−0.2, 1.3)	0.48	0.53	0.75

*DBQ* Dietary Behavior Questionnaire; *MEBS* Mindful Eating Behavior Scale; *BSRS-5* five-item Brief Symptoms Rating Scale; *BDI-II* Beck Depression Inventory II; *PSQI* Pittsburgh Sleep Quality Index; *IPAQ-SF* International Physical Activity Questionnaire Short Form; *MET* metabolic equivalent;. *WHOQOL* WHO quality of life.

*p < 0.05, by Wilcoxon matched-pairs sign-rank test; Data were median (interquartile range).

Δ_1_: median of differences between measures at baseline and day 30 (the end of period 1).

Δ_2_: median of differences between measures at day 30 (the end of period 2) and day 60 (the end of period 2).

Table 2 also showed the impact of app intervention on psychological and behavioral indicators. In control-first group, there was no significant change in eating behaviors, mood, sleep quality, physical activity, and quality of life between control period and intervention period. However, in the domain of “eating in response to hunger and satiety cues”, there was a marginal effect (*p* = 0.056) in intervention period of control-first group. After 30-days app intervention, the increment of eating in response to hunger and satiety cues was higher than life as usual. For intervention-first group, there was no statistically significant difference between two periods among those psychological and behavioral factors.

Table 3 demonstrated the changes in body composition and psychological and behavioral factors after integrating control-first group and intervention-first group into two new groups after completion of intervention with app and control period, they were As usual (n’ = 20) or App (n’ = 20) groups. A decreasing trend of body weight, muscle weight, fat mass, fat%, and weight change ratios were observed in the App group although it did not achieve statistical significance. Conversely, in the As usual group, increasing trend were observed in all body composition parameters. Similar pattern was also observed in MEBS scores. Notably, The App group had a marginal improvement in MEBS total scores than As usual group (Δmean = 0.94 vs. − 1.7, *p* = 0.07), Notably, the improvement in scores of hunger and satiety cues domain was significantly greater in the App group than the As usual group (Δmean = 0.7 vs. − 0.61, *p* = 0.04) (Table 3).

**Table 3:** Comparisons of outcomes in the integrated groups as App intervention and Life as usual.

	As usual (N = 20)				App (N = 20)				Δ_mean_ Between groups
	Baseline	Day 30	*p* Value	Δ_mean_	Baseline	Day 30	*p* Value	Δ_mean_	*p* Value
Body weight (kg)	80.5 (16.8)	80.6 (17.1)	0.50	0.16 (0.8)	80.9 (17.1)	80.5 (17)	0.26	− 0.36 (1.1)	0.17
BMI	30 (6)	30.1 (6.1)	0.51	0.05 (0.3)	30.2 (6.2)	30.73 (7.6)	0.43	0.55 (3.2)	0.24
Muscle weight (kg)	26.7 (5.1)	26.7 (5.1)	0.84	0.02 (0.6)	26.8 (5.1)	26.7 (5)	0.21	− 0.13 (0.5)	0.6
Fat mass (kg)	32 (11.6)	32 (11.6)	0.74	0.09 (1.2)	32.2 (11.7)	32.1 (11.9)	0.44	− 0.12 (1.2)	0.53
Fat percentage (%)	39 (6.7)	39.1 (6.7)	0.72	0.04 (1.3)	39.1 (6.5)	38.5 (6.3)	0.47	− 0.65 (2.5)	0.43
Visceral fat (cm^2^)	145.9 (44.3)	146.5 (47.2)	0.68	0.64 (7.3)	146.4 (46.8)	146.7 (46.1)	0.84	0.29 (5.5)	0.77
BW change (%)				0.2 (1.1)				− 0.4 (1.3)	0.24
DBQ	21 (4.5)	21.2 (4.3)	0.61	0.33 (2.8)	20.9 (4.6)	21.1 (5.4)	0.66	0.33 (2.9)	1.00
MEBS									
Total scores	55.1 (4.5)	53.7 (4.3)	0.12	− 1.7 (4.4)	53.7 (5.4)	54.9 (4.3)	0.27	0.94 (4.6)	0.07
Focusing eating	19.9 (2.3)	19.5 (3.8)	0.84	− 0.1 (2.6)	19.1 (3.3)	19.2 (2.4)	0.52	−0.3 (2.5)	0.67
Eating with awareness	6.4 (2.3)	6.2 (2.2)	0.49	− 0.3 (2)	6.3 (2.5)	6.6 (2.4)	0.14	0.4 (1.2)	0.16
Eating in response to hunger and satiety cues	16.7 (2.9)	16.3 (2.4)	0.29	− 0.61 (2.1)	16.1 (2.3)	16.8 (2.3)	*0.04	0.7 (1.3)	*0.04
Eating without distraction	12 (2.4)	11.8 (3.1)	0.20	− 0.61 (2.2)	12.4 (2.6)	12.2 (3.1)	0.55	0.11 (3)	0.49
BSRS-5	9.8 (3)	9.6 (4.5)	0.82	− 0.11 (3)	9.35 (4.2)	9.38 (3.2)	0.32	− 0.2 (2.8)	0.48
BDI	7.7 (6.6)	8.7 (9.6)	0.66	1.33 (6.7)	9.05 (8.8)	7.9 (6.5)	0.54	− 1.9 (9.2)	0.35
PSQI	6.1 (2.7)	7 (3.4)	0.05	0.94 (1.9)	6.6 (3)	6.4 (3)	0.6	− 0.33 (2.4)	0.07
IPAQ-SF (MET/week)	453.1 (426)	540 (989)	0.31	94 (1010)	931 (1283)	554 (673)	0.60	− 398 (1544)	0.96
WHOQOL									
Total scores	14 (1.9)	14.1 (2.2)	0.54	0.047 (1.3)	14.3 (2.2)	14 (2.2)	0.59	0.024 (1.3)	0.95
Physiological aspect	15.3 (2)	15.8 (2.3)	0.26	0.3 (1.1)	15.5 (2.3)	15.4 (2.5)	0.66	− 0.03 (1.8)	0.84
Psychological aspect	15 (2.5)	14.8 (3.1)	0.69	− 0.4 (2)	15.2 (2.6)	15.1 (2.8)	0.55	0.19 (1.9)	0.40
Social relationship	13.8 (2.8)	14.2 (3)	0.76	0.33 (2.1)	14.6 (3.1)	14.1 (2.7)	0.72	− 0.1 (2)	0.57
Environment scope	15.4 (2.3)	15.5 (2.3)	0.43	0 (1.8)	15.8 (2.6)	15.4 (6.1)	0.51	0.02 (1.5)	0.91

*DBQ* Dietary Behavior Questionnaire; *MEBS* Mindful Eating Behavior Scale; *BSRS-5* five-item Brief Symptoms Rating Scale; *BDI-II* Beck Depression Inventory II; *PSQI* Pittsburgh Sleep Quality Index; *IPAQ-SF* International Physical Activity Questionnaire Short Form; *MET* metabolic equivalent;. *WHOQOL* WHO quality of life.

*p < 0.05; Data were mean (SD).

### App-associated adverse events

There was no App related harmful event observed in each group.

## Discussion

In the present study, we found that the use of CogniNU app had significant impact on reduction in body weight (− 0.55 kg/30 days) and body weight percentage by 0.6% in 30 days. Combined crossover dataset also showed that CogniNU app intervention had positive impact on mindful eating behavior, especially the eating behavior in response to hunger and satiety cues.

The control-first group had no mutual interference between the period 1 (as usual) and period 2 (app). The within group difference in weight reduction and weight change percentage decline displayed statistically significance, showing the effectiveness in using this app for weight control. In intervention-first group, fat percentage decreased in intervention period (-0.4%) but increased in control period after shift back to life as usual. This result indicated that the use of the CogniNU app could indeed effectively reduce fat percentage as well but possibly no prolonging effect after discontinuing app intervention. The re-integrated App group showed improvement in almost all body composition including body weight, weight change percentage, muscle mass, fat mass, and fat percentage. In spite of no statistically significant difference for the result above, this consistent pattern of change in this pilot study still provides us expectation to achieve the goal of health promotion in the advanced version of the app or after recruited adequate participants to exceed the power.

Past studies also indicated similar results to technology-based interventions. Mobile health interventions are viewed as useful tool as low-intensity approaches to weight reduction or adjuncts to traditional strategies^33^. The effectiveness in weight loss has been broadly discussed^7,34,35^. Some metabolic parameters including glycemic control, blood lipids and blood pressure were emphasized as well in other review articles^35,36^. Another randomized controlled trial also revealed the effectiveness of intervention with mobile phone application in not only weight reduction but also BMI, body fat ratio, decrement of waist measurement, and diet habit improvement among individuals with metabolic syndrome^37^. Self-monitoring might be enhanced via technology-based Interventions meanwhile, being related to better adherence^7^.

While it should be noticed during the control period without App intervention, the re-gained fat percentage was observed and may be considered as a sensitive indicator for monitoring change of body composition. Previous research has also reported that among all body components, body fat was the one that increased most during the process of weight gain^38,39^. Some determinants of body composition autoregulation have been suggested to explain the disproportionately rapid rate of fat recovery as opposed to lean mass, for example, the adipose-specific control of adaptive thermogenesis, lean-fat partitioning, as well as collateral fattening and fat overshooting^40^. Moreover, the divergence of successful weight reduction in long-term and short-term results was pointed out from the prior controlled studies^41^. One trial made a notice that the participants reduced weight faster in the first 8 weeks could have more possibilities of showing yo-yo effects which was referred to gaining weight quickly after weights move down and up in a certain of time. Subjects having yo-yo effects eventually did not show significant body weight changes for long-term^42^. In the future, differential effectiveness of short-term or long-term of technology-based weight reduction intervention deserves more investigation. It is worth studying whether extending the days of App use can achieve sustained effect on weight control.

The present study demonstrated the improvement app-based interventions were observed in the aspect of nutrition behavior modification as previous study reported^43^. One randomized controlled trial using mindful app for weight control showed helpfulness in emotional eating and uncontrolled eating^44^. Another acceptance and commitment therapy-based randomized controlled trial on eating behavior also revealed improvement in “Eating for physical rather than emotional reasons” and ” Using food as a reward” for mobile app intervention compared with the control group^45^. Based on the current obesity treatment advises, psychological and behavioral interventions are needed. With psychological and behavioral changes, people with obesity might achieve longer term weight maintenance, promote self-esteem, and improve quality of life^5^.

In our study, both groups of subjects did not show statistically change neither in mood, quality of life, physical activity or sleep quality during intervention and control periods. Compared to previous study^46^, the small number of participants and short duration of intervention could have had an impact on our results. In addition, the subjects were all hospital employees, and the COVID-19 pandemic in Taiwan was the most severe at the time of recruitment. Therefore, the physical and mental stress of medical workers may also be affected by the harsh working environment. As we know, via patient empowerment, shared decision-making and health literacy, mobile application was thought to be effective in patient with depression or anxiety^47^. However, some studies indicated that there was limited response in psychological status with technology-based weight reduction programs^48^. Some application trials for weight management revealed that prominent psychological results were observed primarily in stress reduction only^44,49^. Even though the high comorbidities accompanied by mood disturbance and obesity, studies exploring effective technology-based intervention remained quite limited^50^.

The CogniNU app was the first device to integrate AI technology and cognitive behavioral training program to facilitate weight control from different angles. In this randomized controlled trial, significant improvement in mindful eating behavior in the CogniNU App intervention also allows the team to comprehend the potential of the CogniNU App composing psychological, nutritional, and behavioral change components for promoting physical and mental health promotion. Through the application, users might get the nutrition information related to foods, emotional supports, mindful-eating concepts and other behavioral strategies while dealing with the eating problems. The frequency and consistency of dietary self-monitoring play important parts for the effectiveness of weight reduction^51^. Diet record could be made easier with 3D food picture recognition in this mobile application. Modification to the user experience and the app content had been conducted as well.

### Limitation and strength

The present study had several limitations should be addressed. First, since this was a pilot study, the sample size was small. The participants were hard to be invited during harsh COVID-19 pandemic, especially within hospital setting in Taiwan at that time. Therefore, we used nonparametric analysis for the comparison. We also performed crossover design trying to enhance the statistic power and within/between group comparisons. However, regardless of the above adjustments, the results in the present study should be interpreted very cautiously and a longer intervention period with follow-up could have generated better results for future studies.

Second, the participants were all recruited from the same hospital with similar sociodemographic background and were all relatively healthy adults without systemic chronic disease or major mental illness. The interpretation of the results might not be generalized to different setting simply without modification.

Whereas, this pilot study had several strengths. Though the sample size was small, the retention rate was extremely high. There was no participant dropping out in the midway. Also, the objective data, i.e. body composition, were all measured by the same body measurement tool in the hospital setting. It could be more accurate for the result evaluation for not just self-reported body weight change. And the content in this trial also composed of eating behavior change, which was relatively scarce in the technology-based weight reduction program. Based on the results of this pilot study, to achieve a significant difference in body weight by App intervention, with power = 0.8, alpha = 0.05, the average increase in the control group is 0.16 kg (standard deviation 0.8), the average reduction of App group is 0.36 kg (standard deviation 1.0), delta = − 0.52 for sample size estimation, at least 49 people in each group are required for further advanced controlled trial.

### Conclusion

In conclusion, this randomized controlled trial demonstrated the effectiveness of CogniNU app intervention on health promotion of better body composition, especially body weight, and better mindful eating behavior among overweight/obese people in 30 days. For future study, upgraded advice design, more adequate and diverse participants’ recruitment, and longer intervention period with follow-up were needed to enhance the quality of telehealth and provide more information to apply to general population.

## Supplementary Information

Supplementary Information.

## Data Availability

The authors declare that the main data supporting the findings of this study are available within the paper and its supplementary information files. Extra data are available from the corresponding author upon request.
