# Supplementary material for: Effect of a novel telehealth device for dietary cognitive behavioral intervention in overweight or obesity care
Source: Sci Rep. 2023 Apr 20;13:6441. doi: 10.1038/s41598-023-33238-4 (PMC10116097; doi:10.1038/s41598-023-33238-4)
Supplement: Supplementary file 1 — Supplementary Information. [file 41598_2023_33238_MOESM1_ESM.docx]

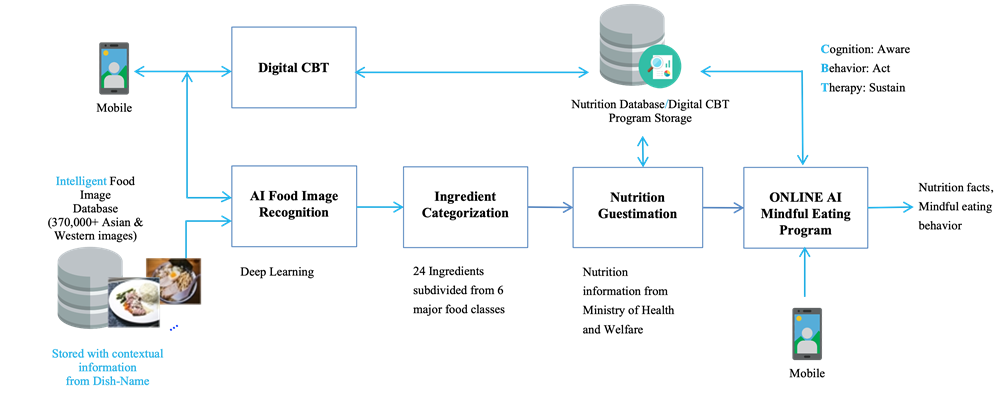


**Supplementary Figure 1.** Block diagram of the CongiNU app.


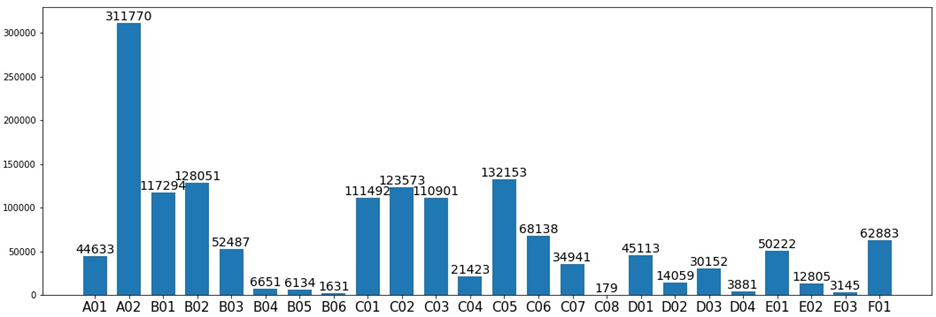


**Supplementary Figure 2.** Distribution of food images by ingredient category in the Intelligent Food Image Database.

**Supplementary Table 1.** Open source datasets of food images.

| Dataset | Provider | No. of images | Food type | Labelling method |
| --- | --- | --- | --- | --- |
| Vireo-Food 172 | City University of Hong Kong (China) | 110,241 | Asian | Dish name & food ingredient |
| Ytower Cooking | Taiwan Food Network (Taiwan) | 23,688 | Asian | Dish name |
| ChineseFoodNet | Media Emerging Technology Center (China) | 185,628 | Asian | Dish name |
| Food-101 | Eidgenössische Technische Hochschule Zürich (Switzerland ) | 101,000 | Western | Dish name |
| ECUSTFD | East China University of Science and Technology (China) | 2,978 | Various | Food name |
| Menu-Match | Microsoft (USA) | 646 | Various (mostly Western) | Dish name |
| UNIMIB2016 | University of Milano-Bicocca (Italy) | 1,027 | Western | Dish name |
| FooDD | University of Ottawa (Canada) & University Istanbul (Turkey) | 3,886 | Various | Food name |
| Food-11 | Ecole Polytechnique Fédérale de Lausanne (Switzerland) | 16,643 | Various | Food category |

**Supplementary Table 2.** Ingredient category.

| Label | Ingredient |
| --- | --- |
| A01 | Rice |
| A02 | Flour products |
| B01 | Pork |
| B02 | Chicken |
| B03 | Beef |
| B04 | Mutton |
| B05 | Duck |
| B06 | Goose |
| C01 | Fruit |
| C02 | Leafy vegetable |
| C03 | Pepo |
| C04 | Flower vegetable/petals |
| C05 | Rhizome |
| C06 | Bean/Nut |
| C07 | Seaweed/Mushroom |
| C08 | Konjac |
| D01 | Fish |
| D02 | Shellfish |
| D03 | Crustacean |
| D04 | Cephalopod/Mollusk |
| E01 | Egg |
| E02 | Tofu |
| E03 | Hard bean curd/Soy milk film |
